# One Health Education Nexus: enhancing synergy among science-, school-, and teacher education beyond academic silos

**DOI:** 10.3389/fpubh.2023.1337748

**Published:** 2024-03-22

**Authors:** Ulrich Hobusch, Martin Scheuch, Benedikt Heuckmann, Adnan Hodžić, Gerhard M. Hobusch, Christian Rammel, Anna Pfeffer, Victoria Lengauer, Dominik E. Froehlich

**Affiliations:** ^1^University College for Agricultural and Environmental Education, Vienna, Austria; ^2^Centre for Teacher Education, University of Vienna, Vienna, Austria; ^3^Austrian Educational Competence Centre for Biology, University of Vienna, Vienna, Austria; ^4^Centre of Biology Education, University of Münster, Münster, Germany; ^5^Division of Microbial Ecology, Centre for Microbiology and Environmental Systems Science, University of Vienna, Vienna, Austria; ^6^Department of Orthopedics and Trauma-Surgery, Medical University of Vienna, Vienna, Austria; ^7^Austria Regional Centre of Expertise on Education for Sustainable Development Vienna (RCE Vienna), Vienna University of Economics and Business, Vienna, Austria; ^8^Department of Education, Centre for Teacher Education, University of Vienna, Vienna, Austria

**Keywords:** One Health, Agenda 2030, Education for Sustainable Development (ESD), Sustainable Development Goals (SDGs), teacher education, biology education, teaching clinic, science education

## Abstract

**Introduction:**

The fact that the daily lives of billions of people were affected by the medical, social, and political aspects of the SARS-CoV-2 pandemic shows the need to anchor the understanding of One Health in society. Hence, promoting awareness and deepening the understanding of the interrelation between human health, animal health, and ecosystems must be accomplished through quality education, as advocated by UN Sustainable Development Goal 4. The often-questioned and discussed measures taken by governments to control the global pandemic between 2020 and 2023 can be seen as an opportunity to meet the educational needs of civil society solutions in multi-stakeholder settings between public, universities, and schools.

**Methods:**

This paper focuses on the integration of One Health principles in educational frameworks, particularly within the context of the higher education teaching framework “Teaching Clinic.” This master-level course in the domain of pre-service teacher education serves as a potent vehicle for facilitating One Health Education, bridging the gap between research, higher education, and schools. Through the presentation of two case studies, this article demonstrates how the Teaching Clinic approach fosters interdisciplinary perspectives and provides a dynamic learning environment for pre-service teachers, as well as for pupils involved in the educational process.

**Results:**

In both cases, the integration of educational One Health school teaching-learning settings effectively enhanced pupils’ understanding of complex topics and engaged them in active learning experiences. Pre-service teachers played a crucial role in developing, implementing, and evaluating these interventions. In Case I, pupils demonstrated proficiency in analyzing data and evaluating mathematical models, while in Case II, the chosen instructional approach facilitated One Health knowledge acquisition and enjoyment among pupils. These results underscore the potential of the One Health Teaching Clinic as a valuable educational framework for enhancing teaching and learning outcomes for pre-service teachers and fostering pupil engagement in socio-scientific One Health-related topics.

**Discussion:**

The discussion delves into the significance of breaking down disciplinary silos and the crucial role of teacher education in promoting a holistic approach to education, emphasizing the intersectionality of One Health Education and Education for Sustainable Development. This article underpins the significance of collaborative efforts across multiple (scientific) disciplines and across secondary and tertiary education levels to reach a nexus. Moreover, it emphasizes the alignment of this approach with the 2030 Agenda, Education for Sustainable Development, and Sustainable Development Goals, highlighting the potential for collective action toward a more sustainable future.

## 1 Introduction: background and rationale for the One Health educational initiative

The ongoing endemic of SARS-CoV-2, the rising incidence and expansion of monkeypox, West Nile fever, Lyme borreliosis, campylobacteriosis, and echinococcosis clearly show that the health of humans, animals, and the environment are closely linked (1). The overarching strategy should focus on key drivers (root causes) of zoonotic diseases at the intersections of the environment, animals, and humans. This nexus involves utilizing expertise in ecology, conservation biology, wildlife biology, and veterinary medicine, alongside emphasizing the need for increased medical and microbiological input into the study of wildlife diseases (2). To put a name to these trans-sectoral and cross-disciplinary dependencies, the term *One Health* (OH) was first coined in 2003–2004 as part of the *One World, One Health: Building Interdisciplinary Bridges to Health in a Globalized World symposium* by interdisciplinary health expert groups associated to the SARS virus and H5N1 avian influenza outbreaks (3). The *Manhattan Principles*, as a consensus-based outcome of the symposium, subsumed a firm recognition for collaborative and cross-disciplinary approaches as well as responses to emerging diseases related to wildlife, food supply, agriculture, environmental conditions, and human health. Over the next several years, the occurrence of growing numbers of zoonotic (animal-human) transmission could be increasingly attributed to changing socioeconomic, ecological, and environmental factors (4) highlighting agricultural intensification (land use) or environmental changes (urbanization) associated with the rapid growth of the world population (5). Although an awareness of the contextual risks and related strategies had already been built up, the emerging pandemics of swine flu in 2009 (Influenza A/H1N1) and the ongoing Middle East respiratory syndrome emerging in 2015 (MERS-CoV) remained predominantly treated within scientific disciplines and epidemiology not reaching public or politic interest at a global scale. Accordingly, formal education generally omits coverage of these topics.

The onset of the SARS-CoV-2 pandemic in early 2020, which affected the daily lives of billions of people in medical, social, and political aspects, was pivotal for the relevance of the original OH concerns (6). Going back to the roots of OH focusing on zoonotic hazards and viral spillovers, the SARS-CoV-2 pandemic provided a worst-practice scenario in human-animal-environment interaction but a key argument as to why the OH approach is indispensable (7). The global situation led to the formulation of the *Berlin Principles*, thereby expanding the *Manhattan Principles* for risks and strategies regarding economic and socio-political contexts (8) with principles 2 and 10 directly addressing education.

A decade after the formulation of the *Manhattan Principles*, initial efforts emerged to think in terms of more concrete research and education frameworks for OH. However, the vast majority of OH education approaches that have emerged to this day primarily focus on training of medical and veterinary professionals, resulting in continued attention within the silos of health and life sciences disciplines (9). Examples are expanding veterinary and medical education in general practice (10, 11), veterinary students’ community outreach (12), OH perspectives in specific medical disciplines (13), SARS-CoV-2 occasion-based education settings (14), and linkages to veterinary ethics education (15). Along the way, some more specific approaches exist to address actual medical challenges related to zoonotic disease mishandling in countries with less developed health systems (16, 17). Additionally, similar cases involve community-based education at institutional and university levels to address localized disease outbreaks (18–20).

For the general population, OH still remains a vague, cross-disciplinary fringe topic elaborated at macro levels by UN-related institutions (WHO, FAO, OIE, UNICEF) (20, 21) or international organizations declarations and efforts (22). A major challenge is raising public awareness of the impact that we/humans are having on the planet (e.g., climate crisis), including on each other (conflicts, wars) potentially compromising our collective sustainability. According to many, the biosphere is now in charge of all life and the world we share, not society nor the economy (e.g., see Stockholm Resilience Centre). Therefore, we argue that OH needs more reasonable and comprehensive frameworks to consider concrete ways for public dissemination. As already stated in 2009, awareness and a better understanding of the relationship between health and ecosystems should be achieved through well-coordinated learning and teaching strategies (23). Related to this, the first efforts ranged from involving professional associations to the inclusion of training opportunities in various professional fields next to early teaching ideas of OH concepts in science classes from secondary schools to universities (9). OH educational materials have been developed by a variety of professionals (veterinarians, physicians, molecular biologists, ecologists, environmental chemists) and made available as open educational resources for schools and higher education institutions (24) as a dissemination strategy. In addition, school- and teacher-based OH efforts exist that focus on the level of surveys and reflection (25).

In this article, we assert that to enable truly effective and widely accessible One Health (OH) Education, it is essential to apply educational frameworks and strategies from inter- and crossdisciplinary backgrounds. We advocate for implementing these approaches in official curriculum programs tailored to the context of intersectional learning within formal education, primarily occurring in school subjects.

OH Education should be taught as early as possible and across a broad range of scientific disciplines (life sciences, humanities, social sciences, arts) and with inter- and transdisciplinary content (26), which means across all school subjects at primary and secondary schools. For early, inclusive, and holistic OH Education, a focus on teacher education is essential as it promises multiplier effects that will eventually reach all levels of education from university to school. Moreover, pre-service teachers (P-STs) demonstrate a compelling set of prerequisites as they undergo training across diverse scientific fields (natural sciences, humanities, and social sciences), alongside their expertise in pedagogical knowledge, content knowledge, and pedagogical content knowledge, preparing them for their future professional roles (27). In the dissemination of knowledge to society, the formal education sector, especially schools, represents one of the most important actors and drivers (28). Universities, with their intellectual capacity, are equally pivotal in redefining their purpose by embracing the fundamental concept of One Health and Wellbeing (OHW), thus optimizing the probability of sustaining all life on this planet while also informing the UN Global Goals for this decade and beyond (29).

## 2 A vehicle for OH education: the teaching clinic

In this article, we present the Teaching Clinic (TC) as a potent vehicle for OH Education.^1^ TheTC combines service-learning (30) and design-based research (31) in the domain of teacher education (32). It builds on service-learning as a link between teacher students’ learning goals and socially relevant work (33) for schools and in-service teachers (I-STs). These teachers often do not have the resources or scientific expertise to meet complex school-based challenges, and this is the gap where the actual service is being provided (34). During the TC-semester, pre-service teachers (master students in Secondary Teacher Education; P-STs) address these school-based challenges defined by I-STs, therefore learning for their later professional work as teachers (35). Research skills are seen as important elements of professionalization within teacher education (36) to establish evidence-based teaching-learning practices (37). This includes skills such as the ability to ask clear questions, critically examine literature, and the ability to collect, analyze, and interpret data. Throughout the whole TC-process, P-STs receive digital coaching from teacher educators and subject matter experts from diverse (scientific) fields and have access to a large platform of relevant, online courses and other resources (38) P-STs can pick their own learning goals and learning pathways (39, 40) and are continuously supported through asynchronous multimodal video feedback (41).

In short, the TC presents itself as an open learning environment with digital and analogous components, with multiple stakeholders, resources, and deliverables that all need to be brought into a coherent pattern by the learners themselves. P-STs need to be versatile in applying these different tools/methods and in adapting to this flexible learning environment. This is demanding, but it also prepares the P-STs for a complex professional environment. The service-learning aspect purposefully promotes the versatile use of tools and methods to adapt to flexible and dynamic real-life-learning environments, which is a key competency for the later working setting of professional teachers.

The TC-approach within teacher education has important implications for the involved stakeholders and beyond. This not only includes the respective P-STs, but also higher education institutions in general, where the so-called “third mission” (the direct impact on society) plays an increasingly important role (42). Therefore, TC shows how state-of-the-art research and OH knowledge can be translated into a teaching-learning sequence (43) that has a direct impact on the teacher, the classroom, and the pupils. This not only narrows the gap between research, higher education and teachers’ real-life working environments, but also aids the important transfer from academia to schools (42). Another important cornerstone within the research professionalization of the P-STs is the participation in the scientific communication of applied research results within the framework of co-authorships (44). Thus, within the scope of TC, research projects have already been published in the sense of peer-faculty co-creation together by P-STs I-STs, and course instructors in national and international peer-reviewed journals (32, 34, 40, 45).

## 3 Learning environment: One Health Teaching Clinic

**Figure 1:**
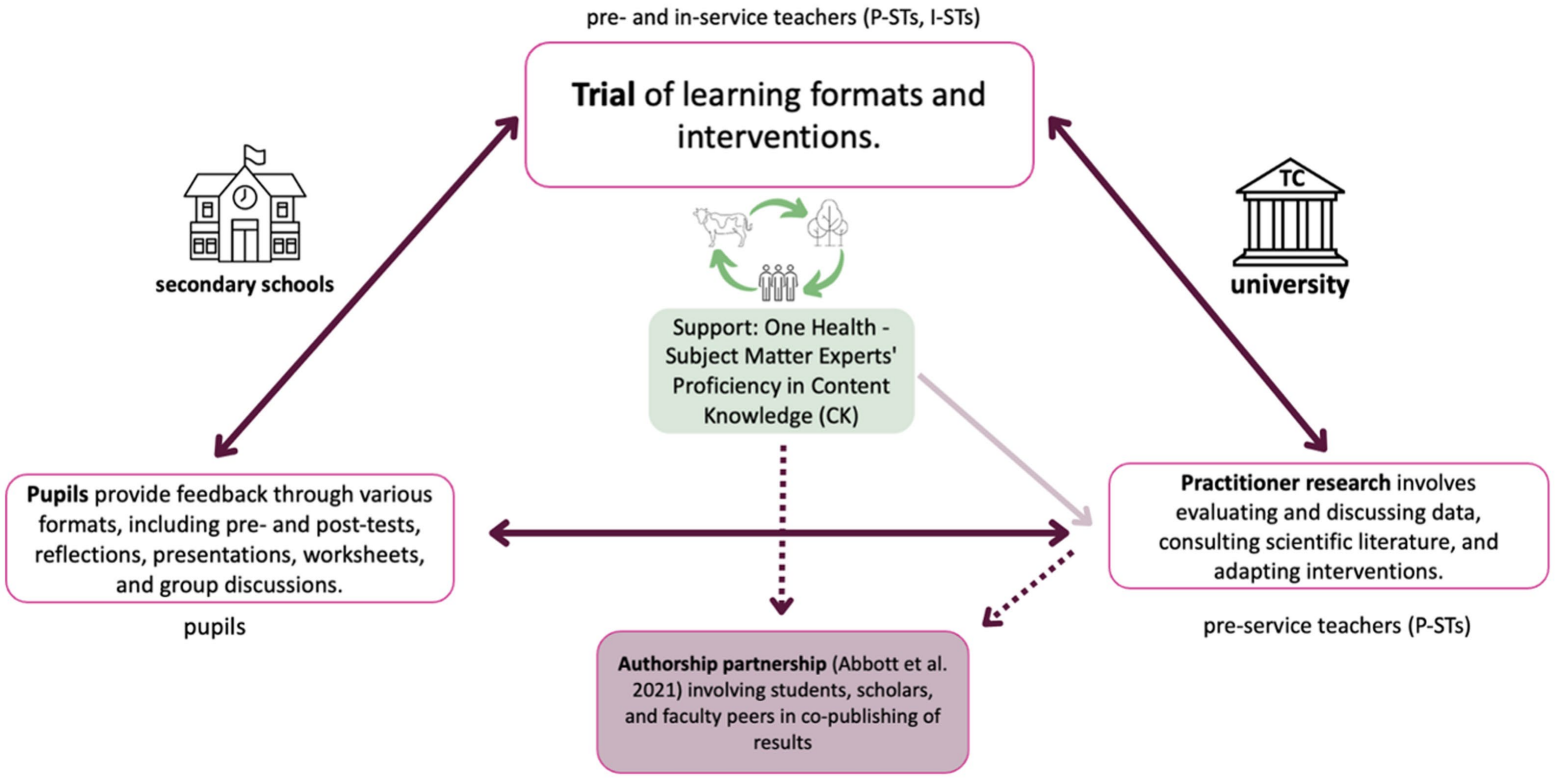
The illustration demonstrates the collaborative utilization of design-based research methods by pre-service teachers in this course, wherein they engage in collective planning, implementation, and evaluation of classroom interventions centered on OH. Working alongside in-service teachers, they are guided by the principles of practitioner research. The TC orchestrates the convergence of four essential stakeholder groups - pre-service and in-service teachers, pupils, teacher educators, and subject matter experts - each assuming distinct roles within the process.

The pedagogical framework of the TC described above has already been implemented to address a wide range of pedagogical issues from different school types and at several tertiary educational institutions. The focus of the *One Health Teaching Clinic* (OH-TC) is based on challenges reported by I-STs at school sites who expressed the wish to integrate the topic of (SARS-CoV-2) pandemic(s) and related risks more into the teaching of different subjects. The framework of OH-TC and involved actors, tasks, and roles is shown in Figure 1.

The selected OH Cases were taught and evaluated with more than 200 pupils ranging from grade 5 to grade 9 by the P-STs themselves; the two first authors, both experienced teacher educators in science education, provided feedback but otherwise did not intervene in the writing process. Thereby, the P-STs did not only pursue the role of *“pre-service”* teachers, but also as co-researchers and pedagogical co-creators (46). In these instances, the P-STs (master-level students) were employed as regular schoolteachers, simplifying the legal aspects as these engagements involved regular classroom teaching rather than intervention studies. This practice aligns with Austria’s context, where schools, facing a shortage of fully qualified teachers, often hire student teachers, even without a Bachelor’s degree or during their Master’s studies.

For each case, the P-STs provide the different objectives, the process (design of intervention study), the outcomes, and a personal reflection statement.

### 3.1.1 Case I: integration of public health knowledge in mathematics lesson

Case I is based on the pandemic-themed board game “Virus Alert in Stayhompton” as a basis, which is available as an Open Educational Resource (OER).^2^ This game was conceived for youngsters aged 12 and above by the Institute of Science and Technology Austria with the Max Planck Institute for Evolutionary Biology. A fictional virus spreading within a city encourages players of the game to investigate and comprehend the various measures that they can employ to manage viral outbreaks. By integrating the game in 7th grade math classrooms, a P-ST wanted to find out what effect the game has on pupils in terms of learning to understand the content related to modeling and statistics, and how pupils use the knowledge to draw their conclusions about the pandemic. For this purpose, the board game was played with the pupils, with an accompanying worksheet serving as a learning and reflection tool (Table 1).

**Table 1:** “Virus Alert in Stayhompton” worksheet for classroom intervention, accompanying the pandemic-themed board game.

1.1 Worksheet “Virus Alert in Stayhompton “Group name: Date:	
Discuss the following questions in your group. One person in your group writes down your answers (in key words or complete sentences)	
Task 1	“Your team of scientists has been able to collect data on the novel virus in Stayhompton. Collect ideas on what this data could be relevant for in the future and what it could be used for.”
Task 2	“Take a closer look at your graph that you created during the game. Try to describe the course of this curve in words.”
Task 3	“Describes the differences between the shape of your curve and that of a direct- proportional relationship.”
Task 4	“Design a poster for the following tasks! (Note: You will present this poster to your colleagues at the end of the lesson).” How would the infection event with the virus proceed if the associated graph represented a directly proportional relationship? What conditions would have to apply to such an infection event? Describe a possible scenario in words and depict it graphically on the poster. Collect arguments as to why you consider such a course of infection in reality to be realistic or unrealistic.

During lessons, pupils actively learned through board games and worksheet exercises. The worksheets helped P-STs to understand pupils’ comprehension and assess their performance, encouraging deep thought from both parties. The final task involved pupils creating poster presentations, and showcasing their ideas while the teacher guided discussions, promoting active participation and diverse problem-solving strategies. This enriched the learning environment, highlighting the importance of engaging and adaptive teaching methods.

**Figure 2:**
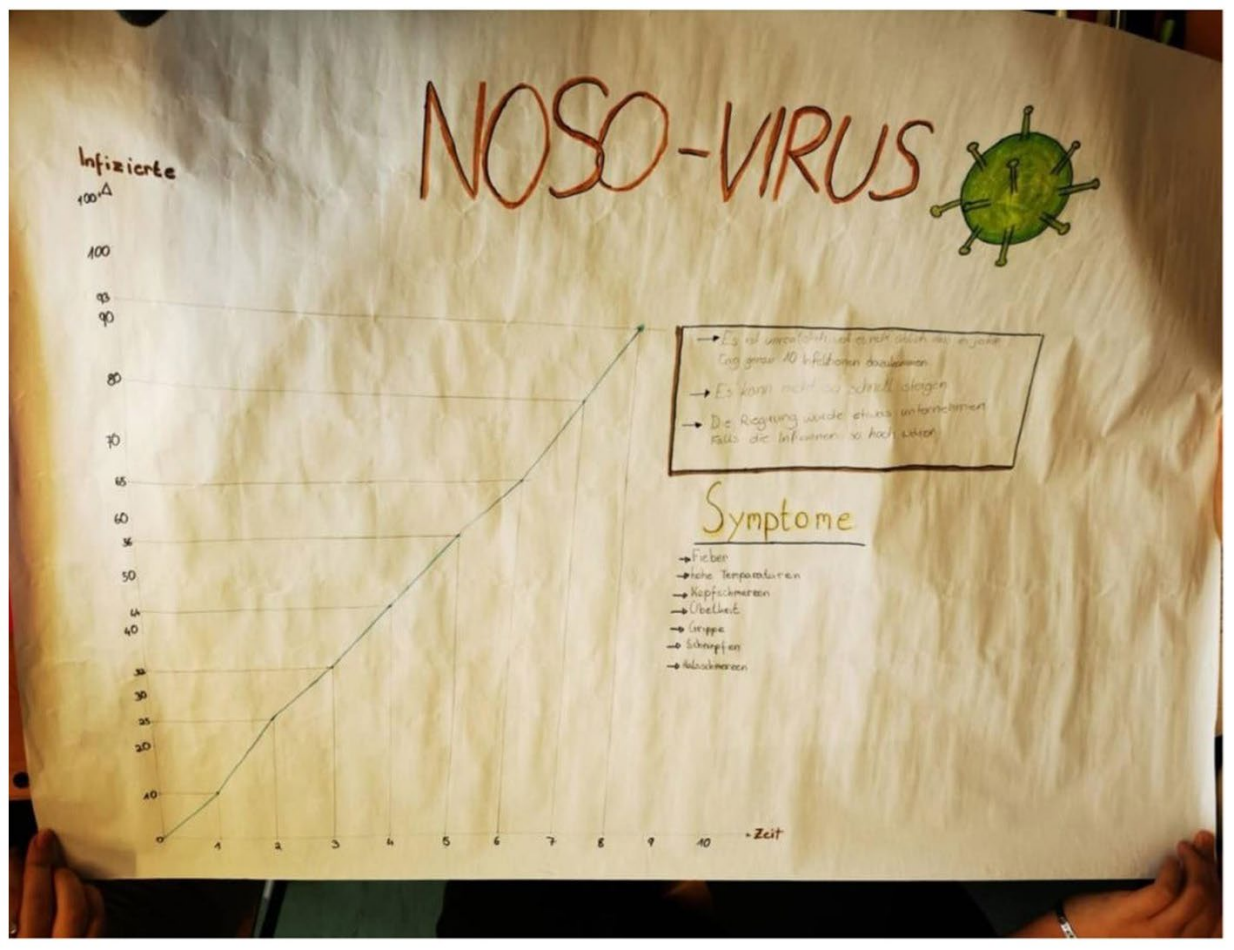
Illustration of a poster (see task4 in Table 1) meticulously designed and presented by the pupils. The posters serve as visual representations of the creative and intellectual efforts put forth by the pupils during the mathematics lesson.

Pupils’ data was coded inductively, with reliability ensured by two independent coders. The results are divided into two parts: group work and presentation. Pupils were asked to discuss potential uses of data generated from a pandemic game. They recognized data as a foundation for making decisions and actions. For instance, Group 1 suggested data could guide lockdown decisions (“We can deduce that the virus spreads very quickly. This allows us to plan for the future. For instance, we can consider whether a lockdown is necessary or not. The data can also be used to prevent the same situation in other places”), while Group 4 proposed using data to prevent widespread hospitalizations (“One could prevent many people from getting sick and needing hospitalization in other cities. The data could be used to impose a lockdown and prevent too many lockdowns in the future. Scientists could invent a medication.”). Pupils analyzed and described the curve pattern of infection data. They described the rise and leveling of the curve, drew comparisons to proportional relationships, and identified differences. Some groups emotionally evaluated the curve’s severity (“Catastrophic, the curve rises quickly. But after a while, it rounds off”). Pupils were given a directly proportional infection model and were asked to contextualize and evaluate it. Groups suggested that real-world infections would not follow such a linear model due to factors like varying interactions and external influences. Within the final task on the worksheet, they concluded that the model was unrealistic and would not hold up in practice (Figure 2; Table 2). Throughout the lesson, pupils demonstrated an understanding of using data for decision-making, describing curve patterns, and critically evaluating mathematical models in real-world contexts.

**Table 2:** Arguments, collectively formulated by two groups of pupils, which comprehensively display their statements concerning the realism of the infection curve within the context of real-world scenarios.

Task 4 (a) Collect arguments as to why you consider such a course of infection in reality to be realistic or unrealistic.	
Group 1	“We came up with a direct proportion with 10 infections per day, meaning one person infects 10 others on the first day. But on the second day, only 10 get infected again, and so on… We do not think that can happen. As more people are infected, there should be more infections happening, not the same amount every day. Eventually, there might be fewer infections when most people are immune, but the same amount every day is not realistic.”
Group 2	“It could happen, like if someone infects multiple others. So, someone infects more, and in our case, it is 5. But it is hard to imagine that the next day only 5 get infected… So, as others have said: It does not work like that in reality. But it would be nice if it did, then we could plan well with hospitals and lockdowns.”

Reflecting on the process and outcomes, the pupils’ recognition of the pivotal role of data in guiding public health decisions during the fragile trust in science was noteworthy. Their understanding was shaped by their experiences with the SARS-CoV-2 pandemic and media coverage, solidifying their belief in the importance of data-driven planning in both scientific and societal contexts. Introduced to linear mapping, the pupils adeptly interpreted curves, linking mathematical concepts to broader societal implications. In the direct proportionality model, they intuitively selected relevant variables, emphasizing the practical significance of mathematical representations for accurate depiction.

The pupils’ emphasis on the relevance of data for justifying future actions, particularly in the context of fragile trust in science, underscored the importance of data as a foundation for planning public health actions, including hospital capacity planning and the necessity of implementing lockdowns. Their adept interpretation of curves using analytical vocabulary and contextual references showcased a mutual understanding, connecting mathematics to real-world implications.

Examining the context references within the direct proportionality model revealed insights into the pupils’ practical perception of mathematical representations as tools for understanding real-world phenomena. Their discussions highlighted the indispensability of mathematical representations for accurate depiction in practical scenarios.

### 3.1.2 Case II: developing One Health knowledge through gamification

Case II is about an educational board game to foster connected thinking in terms of OH for undergraduates. The game was developed by a team of five P-STs from different subjects and was tested in two lower secondary schools in Austria in 2022. The project aimed to find a way to implement OH in school classrooms. Examples from other P-STs from the previous semester were presented at the beginning of the TC and the final decision was to gamify this topic to bring OH closer to the pupils through a playful approach. Gamification in education means to use game elements and game design techniques in an educational context to have “a potential impact on the academic performance, commitment, and motivation of the pupils,” as a systematic review concluded (47).

**Figure 3:**
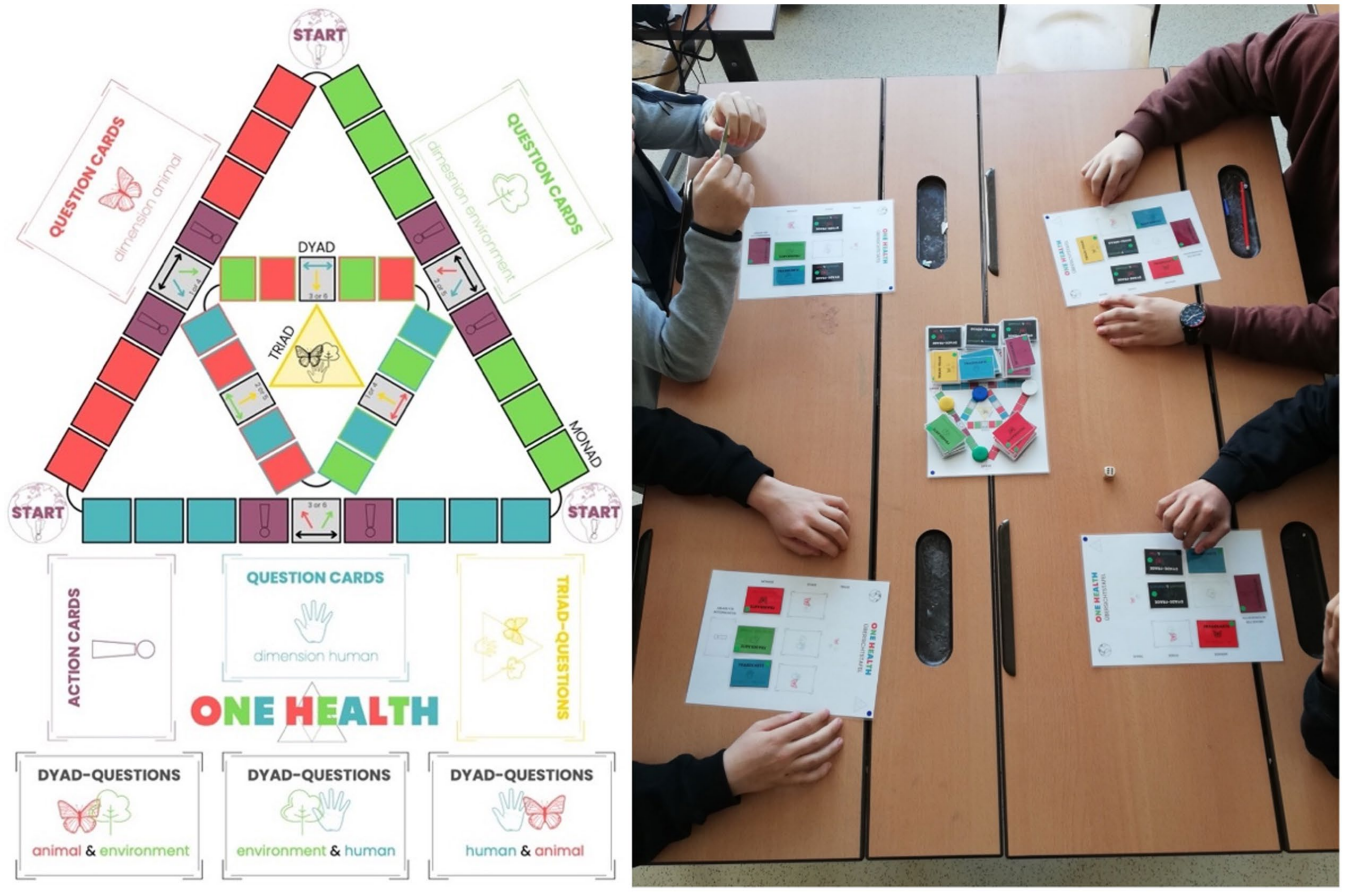
The One Health (OH) board game was designed, enacted in the classroom, and researched by the pre-service teachers as part of the One Health Teaching Clinic (OH-TC) intervention. The rules of the game are similar to the well-known board game ‘Trivial Pursuit,” where a player’s ability to answer popular culture questions leads to victory. However, in the OH board game, players must respond to questions concerning various dimensions of OH. Players advance their game pieces across the board, and the squares they land on determines the subject of the question presented on a card. In contrast to the 6 categories found in “Trivial Pursuit,” such as “History” or “Science and Nature,” the OH board game consists of 7 categories organized within a 3-tier hierarchy of OH, which must be addressed sequentially (dimensions: environment/green, human/blue, animal/red), dyad-questions at intersectional topics (dimensions: animal-environment, environment-human, human-animal) and triad-questions at the OH- dimension of environment-animal-human-intersection (yellow; see left). Every pre-service teacher (P-STs) worked out questions for the game about the three dimensions of One Health. Additionally, action cards (violet) have been included to foster an environment conducive to learning through physical activity tasks within the classroom (48). Three of the P-STs did a pilot test before the game-based intervention with 8 classes (4 different grades included) in lower secondary schools. All pupils (*n* = 124) were asked to do a self-assessment about the concepts of OH. Questions about the fun factor and the willingness of repetition (with family and/ or friends) in their free time were added. The pupils got approximately 100 min to play the game. Finally, the results were analyzed to answer the research questions.

By developing a game (see Figure 3), P-STs wanted to create a playful learning environment where environmental, human, and animal health can be related to each other. Due to this aim, the P-STs explored the current knowledge of the pupils about the connections between these three dimensions and their knowledge gain or improved connected thinking after playing the game.

Based on the analysis of pupils’ responses from lower-secondary classes (*n* = 124), it was found that the implementation of a five-point Likert scale across four categories of interest yielded a significant level of consensus. Notably, the findings demonstrate a substantial level of agreement among the pupils, with 67.4% confirming a tangible acquisition of OH-specific knowledge, 81.5% expressing enjoyment during the gameplay, and 82.3% acknowledging the game’s efficacy as a valuable instructional tool. Furthermore, 58.9% of the participants expressed a keen interest in continuing the game outside of the educational setting (playing the OH board game with friends and families at home). These quantitative results are further substantiated by qualitative data, exemplified by instances where pupils actively expressed an interest in purchasing the game for personal use. Such comprehensive and coherent responses highlight the efficacy and potential of the OH board game as an engaging and informative educational resource.

Reflections by the P-ST note the high level of engagement of the pupils (which was described as a highly rewarding experience) and the game’s usefulness as a didactical tool, especially to introduce the topic and to see the interests of the pupils for this topic during the game. The playful approach does not require a high level of specialist knowledge from the teacher and, on the contrary, can also motivate inexperienced teachers to learn along with the pupils. The topic of OH seemed to be overwhelming for pupils due to its complexity. However, the experiences in class clearly showed that an initial inhibition threshold to deal with this unfamiliar topic decreased when the OH-game was played.

## 4 Discussion

The contributions from diverse fields, as showcased in the previous chapter, underscore the critical role of interdisciplinary perspectives in advancing OH Education also crossing school subject boundaries. The integration of OH principles into educational frameworks holds the potential to cultivate a generation of professionals equipped to address complex global challenges with a holistic and collaborative mindset within their disciplines, and this can be grounded in school first. We are going to discuss the education settings presented in the two cases and then proceed to the disciplinary discussion based on the educational topic.

### 4.1 One Health case studies—linking education and the real world

The OH approach broadens the concept of health beyond just humans to include animal and environmental health within a broader context and up to planetary health (49). When applying Science|Environment|Health (S|E|H) as a pedagogy for complex living systems (50) to the OH approach, the primary focus should be on the scientific aspects of OH. The two case studies illustrate this perspective. Case study I integrates OH and S|E|H, examining the role of science in societal decision-making during the virus alert pandemic simulation and underscores the importance of scientific explanations in understanding complex real-world situations, which are informed by data collection and analysis. Case study II spotlights the multifaceted nature of the OH concept, using the two-eyed seeing heuristic, seeing the world with science eyes and at the same time the actual experienced world (49, 51). Yet, decision-making processes also incorporate non-scientific elements, offering a comprehensive view of OH challenges. Additionally, computer simulations in case study I, while ambitious, would show promise as cognitive tools to deepen understanding of OH complexities (52). In case study II, a game-based method simplifies the OH concept for pupils. By distinguishing human, animal, and environmental dimensions with specific game cards and rules, pupils can view OH issues through the science eyes of the two-eyed seeing framework (49), but while proceeding in the game pupils are learning the dependencies and see the complexity. When applying OH to real-world problems, pupils must merge these dimensions for a holistic interpretation rooted in science but inclusive of other perspectives. In science teaching, a stepwise approach of separating and then combining OH dimensions is helpful. It facilitates the integration of OH perspectives into existing curricula, such as discussing ecosystems’ environmental aspects and later adding human and animal health considerations.

### 4.2 Breaking down disciplinary silos: a holistic approach to education

According to *One Health High-Level Expert Panel* in 2022, OH is a holistic approach aiming to optimize the well-being of humans, animals, and ecosystems, recognizing their interconnectedness. Key principles include fostering equity between sectors, promoting socio-ecological balance, and encouraging transdisciplinary collaboration and stewardship for sustainable solutions (53). Transdisciplinary research, as exemplified in the presented work, aims to unite diverse academic disciplines, breaking down traditional silos between diverse areas (such as clinical research, biomedicine, infectious diseases, social sciences, public health, policy, and environmental health) to collectively address complex OH issues (54, 55). Academic institutions play a crucial role in fostering an environment that encourages interdisciplinary collaboration, necessitating a shift in research approaches and capacity to tackle OH challenges effectively (56, 57). Eradication and control of public health threats related to zoonosis particularly require collaborative efforts of animal and human health services, but also the (active) involvement of the general population, as recently seen within the pandemic progress between the years 2020–2022. Veterinary medicine acts as an initial safety barrier monitoring the health and safety of animals, humans since at least 75% of emerging zoonotic diseases affecting humans originate from diverse animal species (58). Veterinarians task within the OH framework includes the application of different principles in designing integrated surveillance and control measures and developing plans for emerging/re-emerging zoonotic diseases. The help of the public for this task, and therefore a supporting task for education, can reach a higher awareness and sensitivity about the relationships of wilderness and animal food production on a global and local scale (59).

The question for human medicine arises whether the clinical practice of patient care is increasingly shaped by interdisciplinary board-level treatment influenced by economic opportunities (vaccination quotas), political strategies (lockdowns), and decisions made by convened expert committees. The lesson learned from the SARS-CoV-2 pandemic might be that single patient/clinician experiences are closely related to public health perspectives and vice versa. This might give value to human medicine in a broad way but also in subspecialties by developing Building Blocks for One Health Systems that can be used to design and construct solutions (60). Therefore, the doctor-patient dialogue has also a lot of educational aspects, which could be supported by an enhanced OH literacy coming from institutional education in schools and universities: First, educational scientists can aid in understanding patients’ personal (everyday) perceptions of illnesses (61). Second, they can facilitate comprehension of the communication dynamics between doctors and patients (62).

### 4.3 Connection One Health education and Education for Sustainable Development

Rapidly rising knowledge about global change and interwoven problems are increasingly being taught and reflected upon in schools for almost 20 years now (63). However, a growing body of literature in Education for Sustainable Development (ESD) points out that due to the complexity of global challenges pupils find it difficult to translate what they have learned into their own lives (64). Therefore, we are facing an increasing gap between knowledge and action among our youth, which cannot be narrowed by just more conventional knowledge transfer (65). Pupils encounter global problems that seem to be far beyond their control. Due to their complexity, these issues are difficult to grasp and can subsequently create a feeling of powerlessness or eco-anxiety (66). This was particularly evident during the SARS-CoV-2 pandemic and the related interdependencies between mental health and overwhelming situations of young people (67). Therefore, integration of the complex OH topics within disciplinary silos, that are the respective subjects in school, can help to elaborate both: the learning of the content within school subjects, which is a curricular demand, and integrating this into the complexity of the world at the same time to show the embeddedness of disciplinary views. This could even be further expanded in reflecting human centrism toward ecocentrism, as it is already done in educational approaches, which deal with the actual Anthropocene debate (68–70).

All these thoughts so far have implications for the educational system, therefore actual and future teachers have a central function to develop an OH education, embedded in the Sustainable Development Goals (SDGs) and fueled by the educational paradigm of ESD (71).

### 4.4 Teacher education as the paramount cornerstone for a One Health education

Teacher education plays a pivot role in the implementation of inter- and transdisciplinary global educational endeavors and thus for broad dissemination in society. In this context, *Teacher Education for Sustainable Development* is explicitly referred to as a key success factor in achieving the goals of Agenda 2030 and ESD (72, 73). With the 17 SDGs’ overall goals and 169 sub-goals in place, national contexts are to be transferred and established into concrete action plans for all UN member states by the target year 2030 (74). Operating in a coherent context like ESD and the 2030 Agenda with its SDGs, OH education should go far beyond dealing with zoonotic diseases or antibiotic resistance and help researchers and policymakers follow a solid framework that contextualizes emerging diseases and the related public health response. This may not only provide important synergies toward a Roadmap to an OH agenda 2030 (75), but also allows OH to contribute as a vice versa catalyst for achieving the (sustainable development) goals of Agenda 2030 (76). In doing so, the Agenda 2030 SDGs provide a multidimensional set of clearly defined goals that can help contextualize these emerging diseases and identify “OH long-term solutions that are equitable, efficacious, and sustainable” (76). The “two-eyed-seeing” heuristic (77) deals with the level of complexity (as inherent in OH and ESD topics) and embraces involved values as well as different disciplines as an educational rationale for an experienced world. Applying their two-eyed-seeing integrates science and the experienced world, structures the relationship, and helps education to sort out the phenomena. OH Education should be included as early as possible (at the undergraduate level) and with inter- and transdisciplinary content (law, policy and government, economics and commerce, and social sciences, as well as the traditional bioscience approaches including epidemiology, microbiology, and ecology) across universities (78). Teacher education offers a promising starting point as student teachers are trained in a variety of scientific disciplines (natural sciences, humanities, and social sciences) to deal with the everyday problems of society in schools.

### 4.5 Unveiling the nexus: building a collaborative One Health Teaching Clinic network

**Figure 4:**
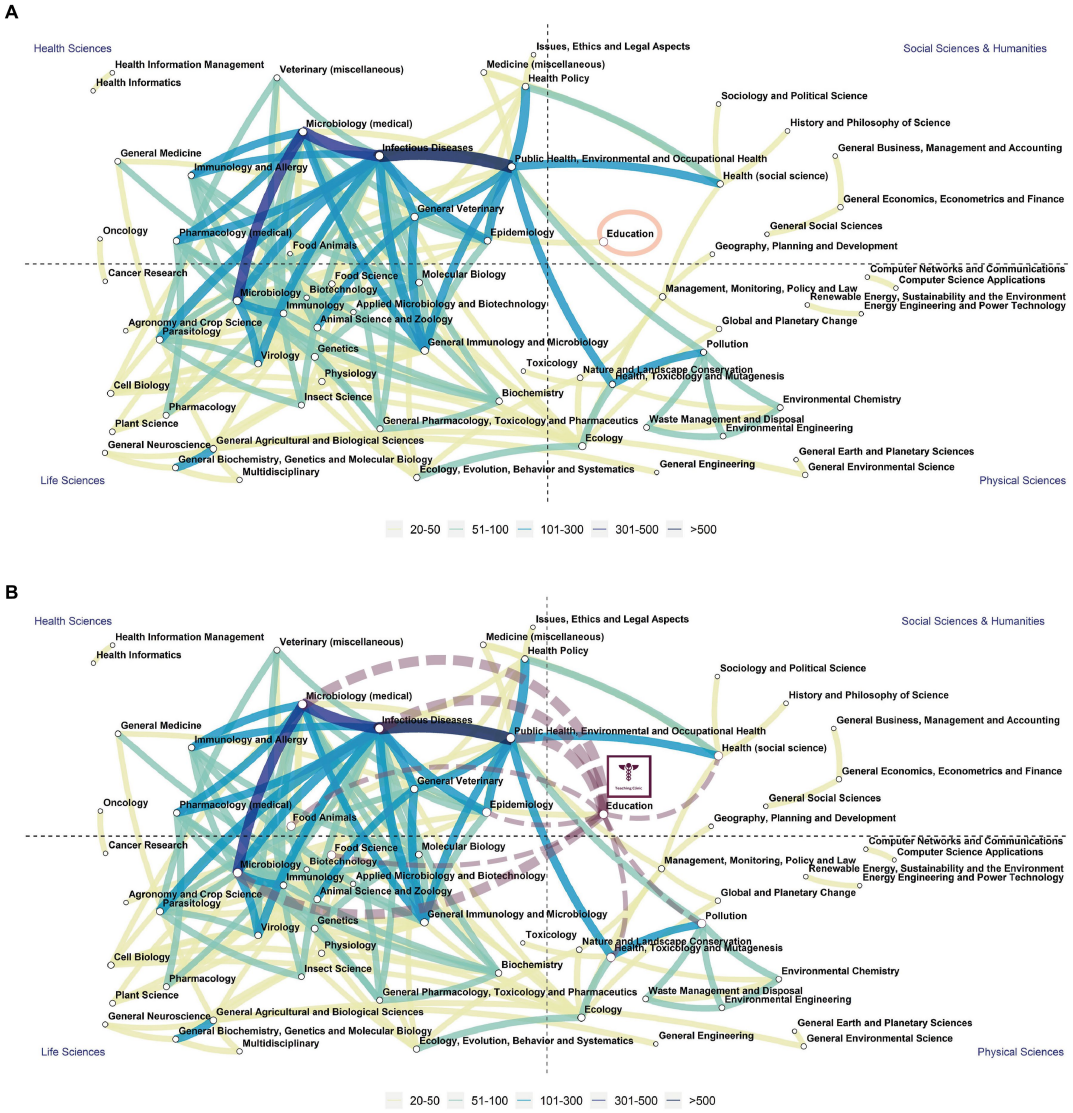
The upper part (**A**, modified with highlighting education) illustrates the network of research fields among OH publications from 2012 to 2021, based on the analysis by Qiang et al. (57). Among over 8,000 publications analyzed, major research areas such as zoonosis, infectious diseases, and microbiology emerged prominently. However, the analysis indicated limited involvement from the Social Sciences & Humanities, notably the education domain. Paths depicted in this illustration denote established connections, adjusted for clarity, with some paths excluded due to fewer than 20 supporting publications. The corresponding modified **(B)** [adapted from Qiang et al. (57)] showcases the *One Health Teaching Clinic Network*. This network is specifically designed to foster collaboration and networking across various scientific fields and sub-disciplines. Its overarching purpose is to bolster the presence of the *Social Sciences and Humanities* while bridging the gap between different fields, aiming to integrate them more cohesively. Adapted from Figure 4 in Qiang et al. (57), Copyright Elsevier (2022).

This final chapter emphasizes the paramount importance of effective OH Education. Even amidst the ongoing endemic, *Social Sciences & Humanities*, particularly *Education*, persistently lacks adequate representation within the *One Health Education Nexus* (see Figure 4A) (57).

To bridge the existing gap between disciplines, it is imperative to translate the invaluable findings gleaned from the case studies presented herein. This pivotal translation of insights serves as one of the fundamental pillars underpinning the envisioned *One Health Teaching Clinic Network*—an interactive teaching and learning process involving stakeholders within the TC (Figure 1). Alongside this, fostering interdisciplinary collaboration by breaking down entrenched disciplinary silos stands as a critical second pillar.

These two pillars are complemented by two others: creating crucial synergies with Education for Sustainable Development and cultivating OH Teacher Education as a gateway to broader society. These four pillars collectively form the foundational framework of the *One Health Education Nexus*, as proposed by the authors. Their significance goes beyond fortifying OH Education; they are instrumental in facilitating collaboration and networking across diverse scientific fields, as depicted in Figure 4B.

The imminent launch of the *One Health Teaching Clinic Network* in the winter semester of 2024/25 represents a significant stride forward. This initiative aims to unify several teacher training institutions and experts from the One Health domain in the DACH region. Its primary objective is to lay the groundwork for a pilot program in formal OH Teacher Education.

This network is not confined to strengthening OH Education; it is a catalyst for collaboration and networking across various scientific fields, envisioning a visionary educational structure. By uniting educators, practitioners, and experts from diverse OH domains, this initiative holds the potential to spearhead a paradigm shift in interdisciplinary cooperation and knowledge exchange. The ultimate goal is to facilitate transformative OH Education, paving the way for a truly integrated and collaborative approach that transcends disciplinary boundaries.

## Data availability statement

The raw data supporting the conclusions of this article will be made available by the authors, without undue reservation.

## Ethics statement

Ethical approval was not required for the study involving humans in accordance with the local legislation and institutional requirements. Written informed consent to participate in this study was not required from the participants or the participants’ legal guardians/next of kin in accordance with the national legislation and the institutional requirements.

## Author contributions

UH: Conceptualization, Formal analysis, Investigation, Methodology, Project administration, Visualization, Writing – original draft, Writing – review & editing. MS: Conceptualization, Formal analysis, Investigation, Writing – original draft, Writing – review & editing. BH: Formal analysis, Writing – review & editing. AH: Formal analysis, Writing – review & editing. GH: Formal analysis, Writing – review & editing. CR: Formal analysis, Writing – review & editing. AP: Formal analysis, Investigation, Writing – review & editing. VL: Formal analysis, Investigation, Writing – review & editing. DF: Formal analysis, Methodology, Resources, Writing – review & editing.

## Footnotes

^1^For an overview of the general teaching clinic concept in teacher education, visit https://teachingclinic.org.

^2^ https://ist.ac.at/en/education/ista-for-kids/virus-alert/
